# Studies on structure property relations of efficient decal substrates for industrial grade membrane electrode assembly development in pemfc

**DOI:** 10.1038/s41598-018-30215-0

**Published:** 2018-08-14

**Authors:** Sri Harsha Akella, Ebenezer D., Sai Siddhardha R. S., Alkesh Ahire, Nawal Kishor Mal

**Affiliations:** Material Science and Technology, Innovation Center, Tata Chemicals Limited, Pune, India

## Abstract

Electrode fabrication and membrane electrode assembly (MEA) processes are critical steps in polymer electrolyte membrane fuel cell (PEMFC) technology. The properties of decal substrate material are important in decal coating technique for efficient transfer of catalyst layer. In the present study, MEAs are fabricated in decal method using 6 different decal substrates among which polypropylene (PP) is found ideal. Morphological, thermal, spectroscopic and sessile drop measurements are conducted for 6 decal substrates to evaluate the thermal and physicochemical properties. Studies indicate PP is thermally stable at hot-press conditions, having optimal hydrophobicity that hinders the coagulation of catalyst ink slurry cast. The pristine PP film has been identified to showcase 100% transfer yield onto the Nafion membrane without contamination and delamination of catalyst layer from membrane. The PP based MEAs are evaluated underconstant current mode in a hydrogen-oxygen fuel cell test fixture. The performance is found to be of 0.6 V at a constant current density of 1.2 A.cm^−2^. Besides, the cost of PP-film is only 7.5% of Kapton-film, and hence the current research work enables the high throughput electrode fabrication process for PEMFC commercialization.

## Introduction

Gross domestic product (GDP) of developing countries benefit with close causation to the energy production and its consumption^1,2^. Increased energy consumption and forefront global issues have led to renewed focus on energy generation and storage. Henceforth, energy security is a promising area of research to public and private governing bodies. The upcoming decades are full of quest for renewable energy conversion and storage devices. Hence hybrid systems that comprises fuel cells, batteries, super capacitors and hybrid of these act as pivot for this energy sustainability^3^. In particular, fuel cell plays a critical role for rural as well as sub-urban electrification, and as off-grid back up power source in telecom industry for many countries^4–6^. In fuel cell system, balance of plant (BOP) and stack development encompass 71% of the fuel cell system cost^7^. Therefore, it is of fundamental challenge for scientists to decrease the costs of stack components without compromising the electrochemical performance. Essentially, fuel cell stack is engineered with critical components such as membrane electrode assemblies (MEAs), bipolar plates, current collector plates, endplates etc. MEA is considered as the heart of the fuel cell and is the epicentre for electrochemical reactions that generate electricity^8,9^. Therefore, MEA fabrication with precision engineering is of fundamental interest and many routes have been put forth considering cost, performance and other entities^10,11^.

Among the various techniques of MEA development, studies have shown that decal technique has improved efficiency over spray and other conventional coating techniques^12,13^. In decal method, the substrates are coated with the catalyst inks, dried and later transferred to a membrane of interest to fabricate the MEA. Conventionally, polytetrafluoroethylene (PTFE) and polyimide (Kapton) films are well known decal substrates for fabricating MEA by decal method. The prominent features of the decal substrates are: (1) The substrate must be chemically inert to the coated catalyst layer, (2) Substrate should not also poison the catalyst during the hydraulic hot-press conditions of the MEA, (3) The hydrophilic nature of the substrate helps to avoid the formation of micro islands of the catalyst ink^14^. (4) The substrate thickness must be less than 50 micron to gain an advantage of easy peel off, after the hot-press of MEA. (5) The substrate must be cost effective for commercial scale up.

**Figure 1:**
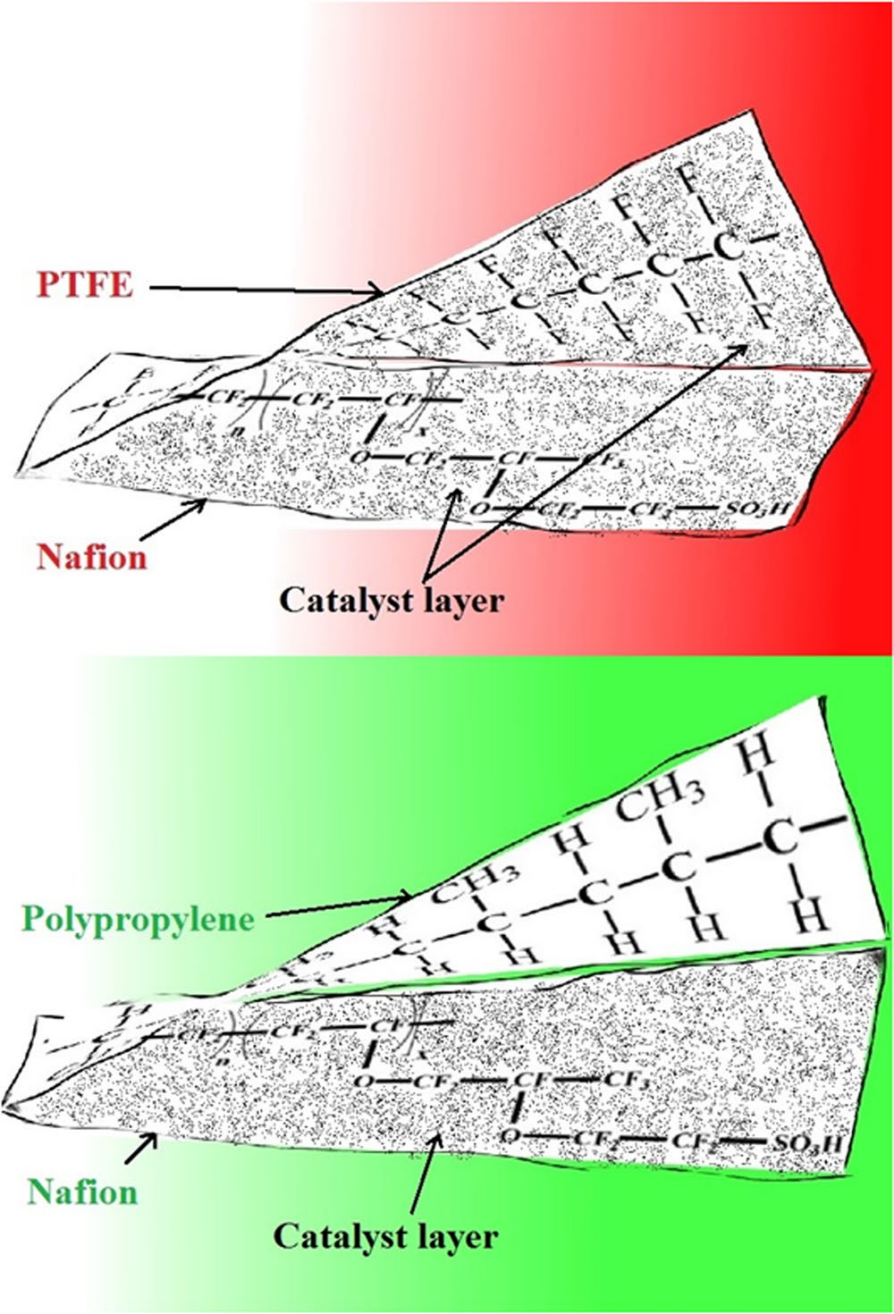
Schematic representation of physicochemical interaction between the PP and PTFE decal films with Nafion based catalyst layer after hot-pressing of the MEA.

It is necessary to understand the structural properties of the decal substrate/films to elucidate the chemical interaction between ionomer/Nafion and the decal substrate. When PTFE is used as the decal substrate, due to increased chemical interaction between PTFE and Nafion only partial catalyst transfer has been observed during the hydraulic hot-press subjection (Fig. 1). Irmawati *et al*.^15^ has concluded that PTFE cloth and film furnish only 90% catalyst transfer. Similar works of Shahgaldi *et al*.^16^ has observed only 85% catalyst transfer yield using PTFE as substrate. Furthermore, it has been studied that fluorinated ethylene propylene substrate has the best catalyst transfer efficiency. To achieve this an additional coat of Nafion over the catalyst coated decal substrate with a loading of at least 0.2 mg. cm^−2^ is necessary prior to hot-pressing with the membrane (Table 1)^16^. However, Sasikumar *et al*. has disclosed that more than 30 wt.% Nafion ionomer loadings will block the meso-nano pores of the catalyst layer which hinders the performance of fuel cell^17–19^. Other decal substrates such as aluminium foil, stainless steel, perfluoroalkoxy polymer, polytetrafluoroethylene, Kapton HN (Polyimide), and Kapton FN (80% polyimide and 20% fluorinated ethylene propylene (FEP)) etc. have also been considered with partial catalyst transfer and low transfer efficiency (Table 1). Therefore, it is vital to understand the chemistry of the decal substrates along with economics to achieve complete catalyst transfer for an industrial MEA development. Many decal substrates are reported with complete catalyst transferability such as PP, Kapton HN, polyethylene(PE) films, however they are only qualitative claims^20^. Furthermore, to the best of our knowledge, no literature is available related to ideal decal substrates for the complete transfer of catalyst layer to the Nafion.

**Table 1:** Type of decal substrate and catalyst transfer ability.

Substrate	Catalyst layer transfer percentage	
PTFE	69	^16^
FEP	42	
Aluminium foil	56	
Kapton FN	50	
Kapton HN	25	
Steel substrate	27	
FEP with 0.2 mg.cm^−2^ Nafion outer layer	100	
PTFE cloth	90	^15^
PTFE Film	90	
Aluminium foil	90	

In the present work, an extensive study has been carried-out with a quest to find outcost effective, 100% catalyst transferable decal substrate. Hence PP, Low-density polyethylene (LDPE), Silicone coated polyethylene terephthalate (Si-PET), Polytetrafluoroethylene/Teflon (PTFE), Reinforced-polytetrafluoroethylene (RPTFE), Kapton films has been selected. The substrates are evaluated for their properties that are relevant towards MEA fabrication. During MEA fabrication, a blend of physicochemical properties such as thermal stability, chemical inertness of the substrate plays a key role. We identify PP as complete catalyst transfer decal substrate. The other benefits with PP use are low cost, non-poisoning of the catalyst, high electrochemical performance upon MEA fabrication. The cost of PP is just only $ 18.m^−2^ and for Kapton it is $ 240.m^−2^, this research therefore drastically brings down the cost of MEA fabrication. Since MEA is the heart of the fuel cell, this is a giant leap for PEMFC commercialization. The detailed scientific aspects of the decal substrates and their catalyst interactions have been discussed in the subsequent sections.

## Results and Discussion

The complete catalyst transfer over the Nafion after the hot-press is a crucial step. The MEA fabrication efficiency is evaluated in terms of percentage transfer of the catalyst ink and catalyst rejuvenation post fabrication. The percentage transfer of the catalyst is evaluated for different substrates using hot-press and the tabulated in Table 2.

**Table 2:** Different parameters that affect the decal based MEA fabrication using different substrates.

Substrate Name	Thickness of substrate (micron meter)	Catalyst layer transfer percentage	Poisoning	Scalability
PP	30	100	×	✓
LDPE	30	100	✓	×
Si-PET	50	95	×	×
Kapton HN	15	92	×	✓
PTFE	90	64	×	×
RPTFE	220	72	×	×

PP has 100% transferability than any another conventional substrates such as Si-PET, Kapton, PTFE and RPTFE. It is evident from the tabulated data, because of thermal and chemical stabilities during hot-press step, the thin PP film are effectively used to transfer the catalyst. During hot press, the use of Teflon as substrate has provided low percentage of catalyst transfer towards the Nafion membrane. Further, in terms of the case of manufacturing & scalability, PP and Si-PET substrates are cost effective films at a commercial scale. Conversely, other decal substrate due to hydrophobicity forms isolated/islands of catalyst ionomer segregation causing hot spots over the MEA, this directly diminish the quality of the MEAs^14^. Hence, surface chemistry, structural and thermal stabilities of selected substrates are crucial and discussed in the latter sections.

A skin tattoo inspired decal substrates peel off from the Nafion membrane after hot-press. The Fig. 1 shows the schematic depiction of interactions of PTFE, PP decal films with catalyst layer. The bottom segment presents: partial transfer of catalyst layer with PTFE, whereas top segment presents: complete transfer of catalyst layer onto the Nafion using PP decal substrate.

### Calorimetric analysis

**Figure 2:**
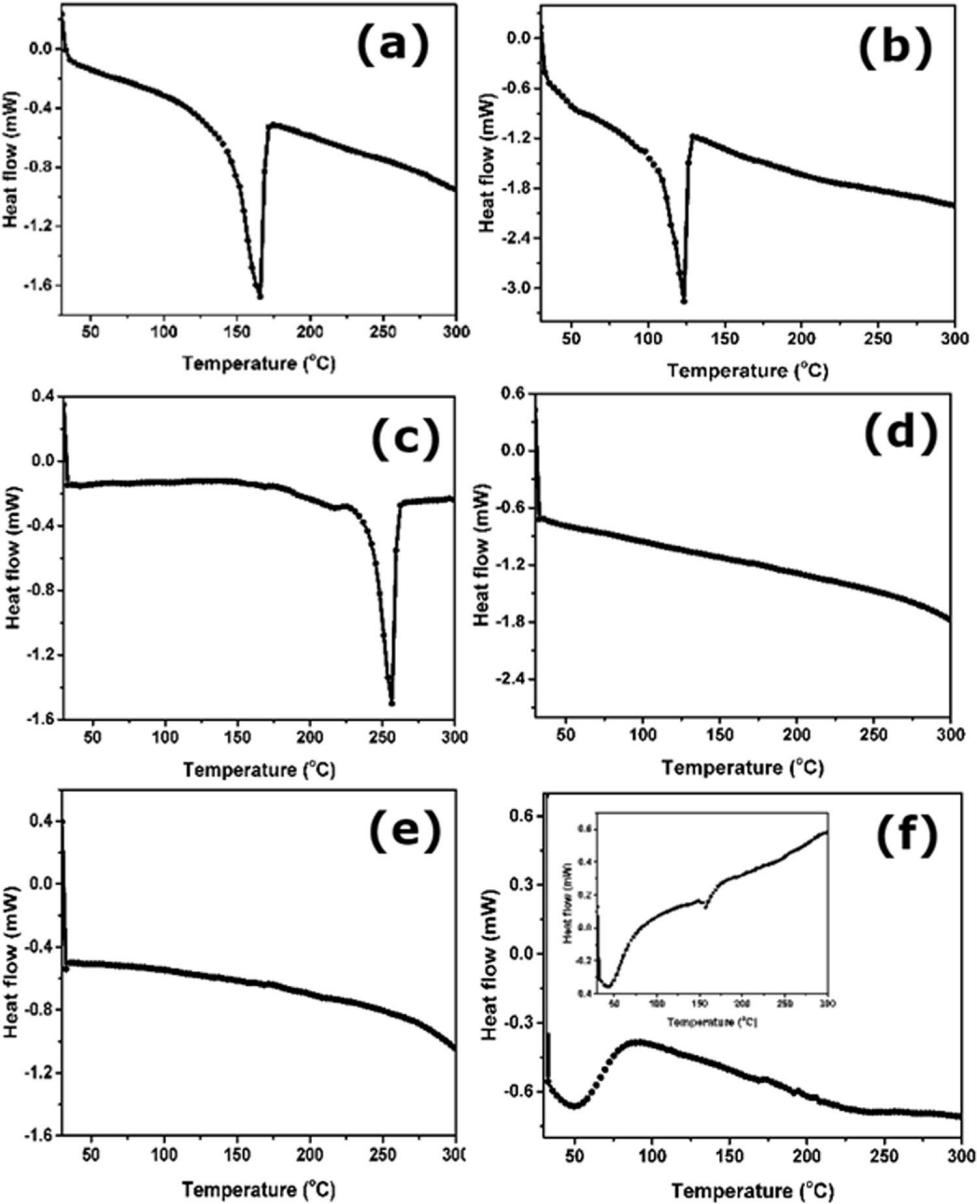
DSC curves for different substrates (**a**) PP, (**b**) LDPE, (**c**) Si-PET (**d**) RPTFE (**e**) Teflon (**f**) Kapton, inset shows DSC profile of Nafion.

During hot-press conditions of MEA, thermal decomposition of the decal substrate generate many products and side products that in turn may poison the catalyst/electrode. Hence, thermal behaviour of these substrates are evaluated using calorimetric studies^21,22^. Their profiles are shown in Fig. 2(a–f). The melting point of LDPE is ~122 °C, which is at hot-press condition/temperature of the Nafion based MEA. Though, LDPE transfer yield is 100%, the catalyst got poisoned. Conversely, PP films exhibiting minimal phase change till 140 °C and hence the PP substrate is chemically inert with catalyst layer. Figure 2(d–f) show calorimetric profiles of the RPTFE, Teflon, Kapton, and inset – Nafion films and it is discernable that these films are stable, however very low yield of catalyst transfer is observed due to binder-substrate interaction at high temperature and pressure. To understand these interactions, structure-property relations are evaluated using contact angle and spectroscopic studies^23–26^.

### Spectroscopic analysis

**Figure 3:**
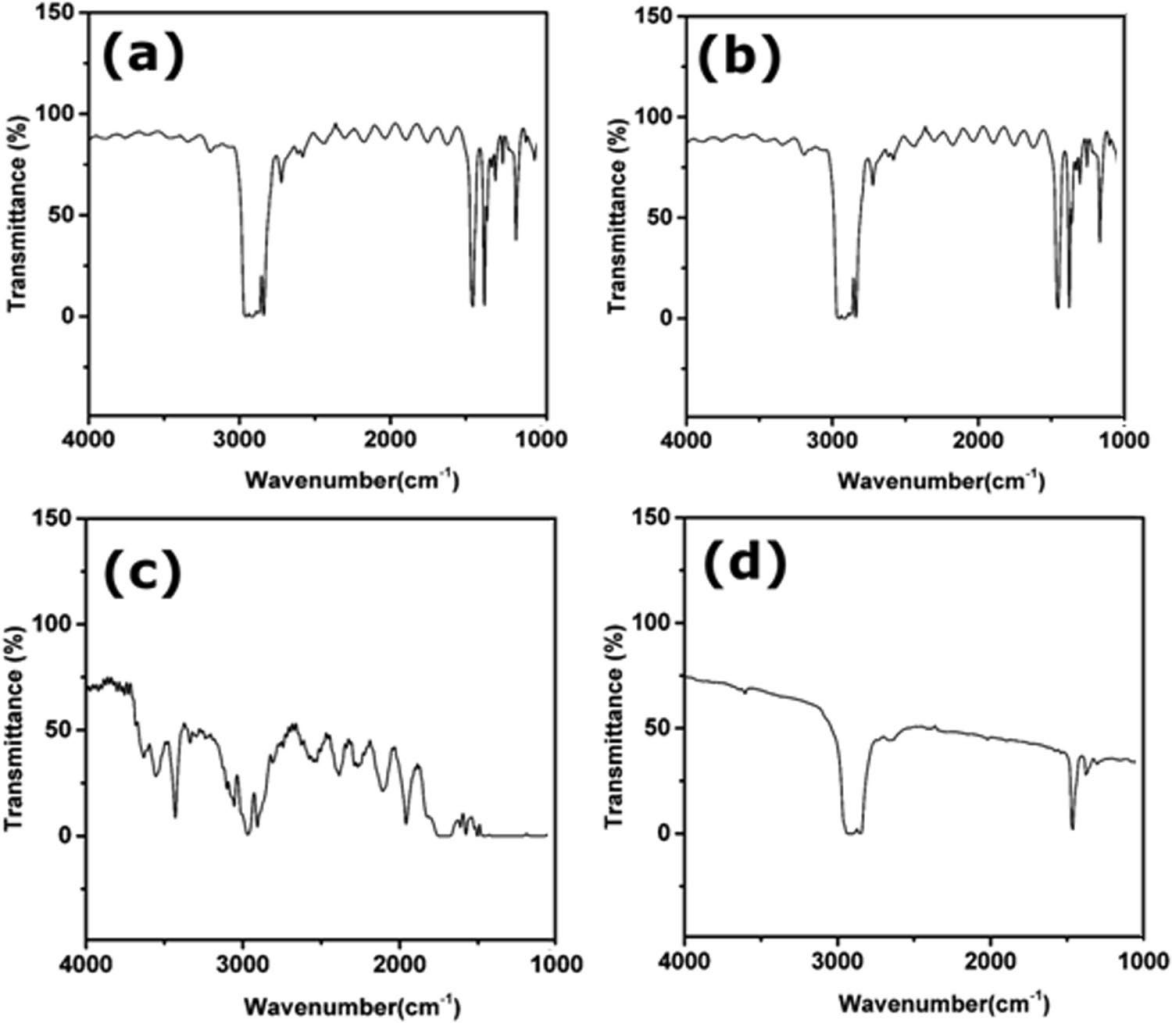
FTIR measurement of different substrates (**a**) PP, (**b**) Cellophane tape, (**c**) Si-PET (**d**) LDPE.

**Figure 4:**
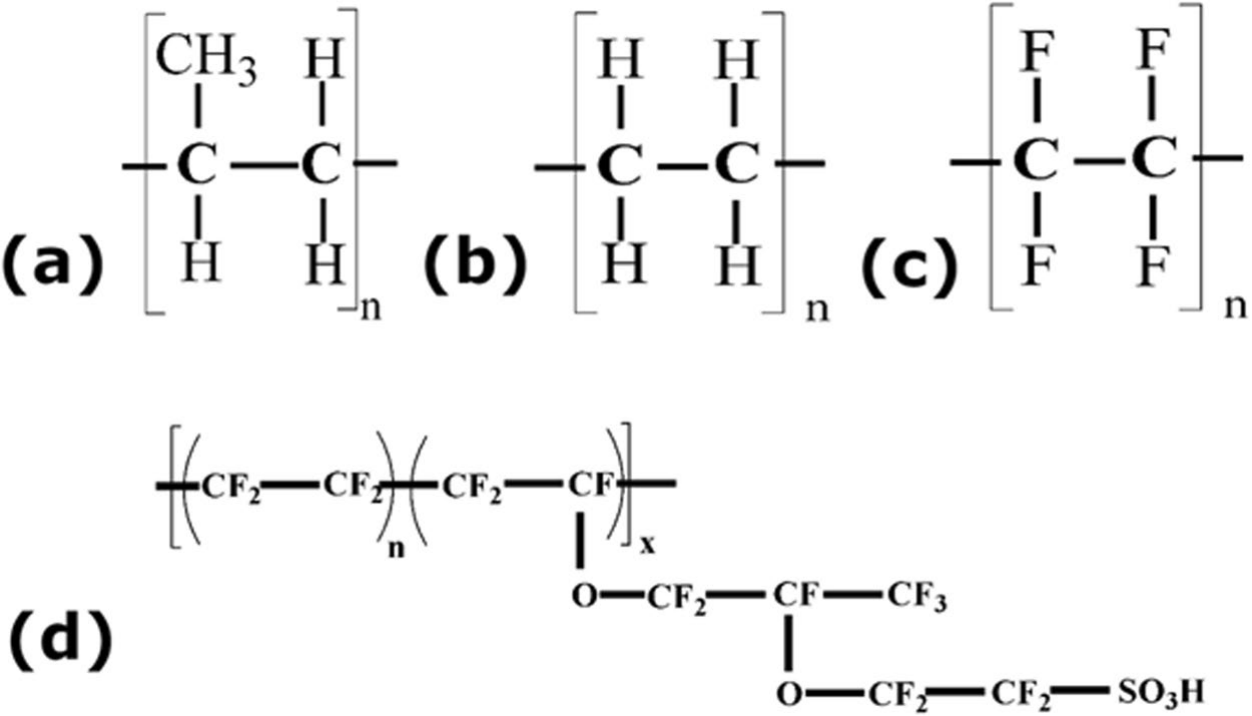
Chemical structures of (**a**) Polypropylene (PP), (**b**) Polyethylene (PE), (**c**) Polytetrafluoroethylene (PTFE), (**d**) Nafion.

FTIR profiles of PP and cellophane substrates in close resemblance and are presented in Fig. 3(a) and (b). The long band in Fig. 3(a) from 2800 to 3000 cm^−1^ is C-H stretching vibration, the peaks at 1454 cm^−1^ and 1377 cm^−1^ are bending absorption of CH_2_ and CH_3_ respectively, these peaks are characteristic of PP material^26^ (refer Fig. 4(a) for the polypropylene structure). The weak vibration at 1377 cm^−1^ implies the material is low density polyethylene Fig. 3(d)^27^. The silicon coated polymer (Si-PET) represent many acetyl and hydroxyl functional groups that are responsible for partial interaction with Nafion causing in-complete transfer of catalyst after hot-press as shown in Fig. 3(c) (refer Fig. 1 for pictorial representation). PTFE and RPTFE are thermally stable but not able to transfer the catalyst layer completely because of chemical interaction with Nafion (Fig. 4(d)). FTIR spectrum is recorded after hot-pressing the plain decal PP substrate without the catalyst ink. No additional peaks have been observed in the spectrum post hot-pressing. This also indicates that there is no change in surface chemistry of the PP substrate. Therefore PP film is an efficient decal substrate without additional Nafion loading over the decal catalyst layer.

Chemical structure of polypropylene, polyethylene and Nafion are as follows:

### Contact angle measurements

**Figure 5:**
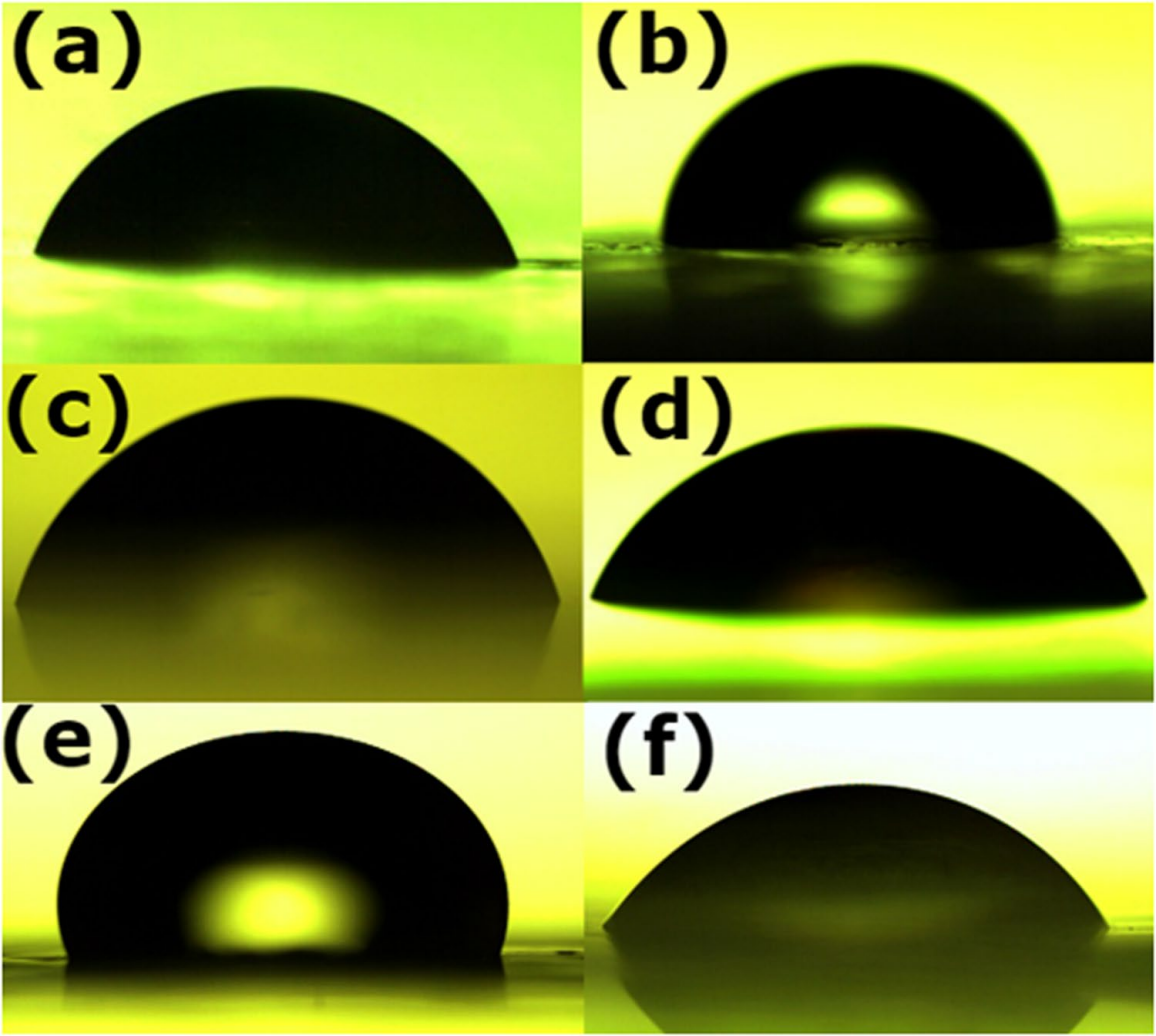
Contact angle measurement with water using Olympus microscope for (**a**) PP, (**b**) LDPE, (**c**) Si-PET (**d**) Kapton (**e**) RPTFE (**f**) Teflon.

Contact angle is measured using a confocal microscope to study the substrate surface characteristics^28^. The contact angle images of different decal substrates with water are presented in Fig. 5. The hydrophilicity of the substrate tends to uniformly spread the coated catalyst ink. On the other hand, hydrophobicity tends to coalesce the ink slurry forming micron sized islands of catalyst layers over the substrate after the coating^14^. From this proposed postulate, we infer that the catalyst ink can be spread uniformly by choosing that right substrate. During the hot-press, this phenomenon indirectly controls the transferability of catalyst layer to the Nafion. PP has contact angle of 90^o^ (refer Table 3) and therefore displays optimal hydrophobicity with water and leads to decreased ionomer/catalyst ink aggregation. The RPTFE, however, is highly hydrophobic with contact angle of 106°. This has resulted in partial catalyst transfer and micro island formations. The substrates Teflon 84°, Si-PET 74° and Kapton 70° have only shown partial catalyst transfer.

**Table 3:** Water drop contact angle studies over different substrates.

Substrate	Contact angle (Degree)
PP	92
LDPE	87
Si-PET	74
Kapton	70
Teflon	84
RPTFE	106

It is important to note that substrate should not interact with Nafion in order to transfer the catalyst completely.

We have reported complete transferability of catalyst with ultra-low cost decal substrate without any chemical treatment. From the physicochemical studies of different substrates, PP films are identified as suitable decal substrates for commercialization of MEAs.

**Figure 6:**
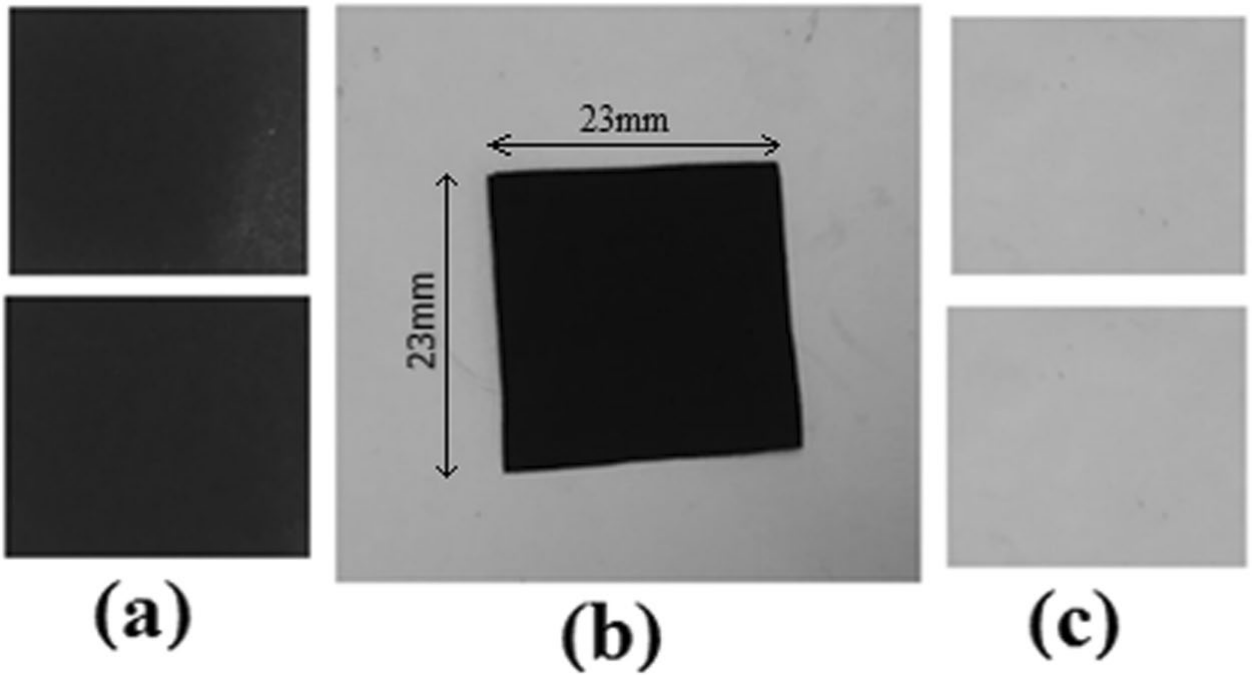
(**a**) Decal substrate with catalyst coating before hot-press (**b**) Geometry of the MEA prepared using decal method via hot-press (**c**) Decal substrate after complete transfer of catalyst layer on to the Nafion after hot-press.

The complete transfer of catalyst from PP substrate after hot-press has been clearly presented in 6. Decal substrates having catalyst layer is presented in Fig. 6(a), MEA fabricated after hot-press is shown in Fig. 6(b), the peeled, decal film before and after hot-press is shown Fig. 6(c) indicating complete transfer.

**Figure 7:**
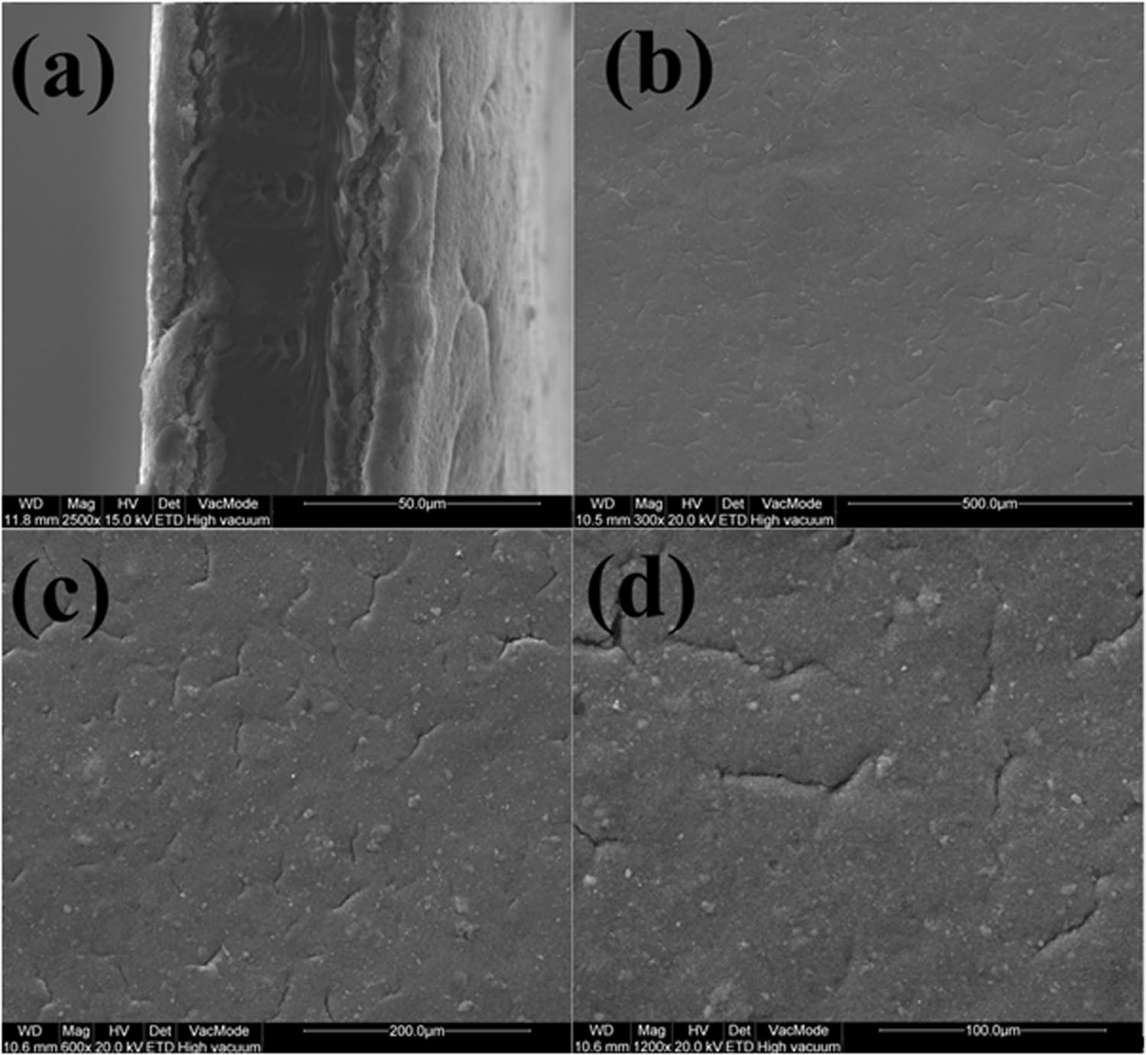
SEM images of (**a**) Cross section of MEA, Surface morphologies of catalyst layer on the MEA at different magnifications (**b**) 300x (**c**) 600x (**d**) 1200x magnification.

The catalyst layer on the either sides has been uniformly distributed over the polymer electrolyte membrane as shown in Fig. 7(a). The electrode has uniform mesoporous cracks all over the surface as observed in Fig. 7(b). These mesoporous cracks are as a result of Nafion quenching after the hot-press. These cracks aid in enhanced permeation of reactant gases and further lead to increased catalyst utilization and subsequently good PEMFC performance. The SEM images of the catalyst layer with improved magnification are presented as Fig. 7(c,d).

### Single cell performance evaluation

The PP based MEA is evaluated under constant current mode in single cell, close cathode condition.

**Figure 8:**
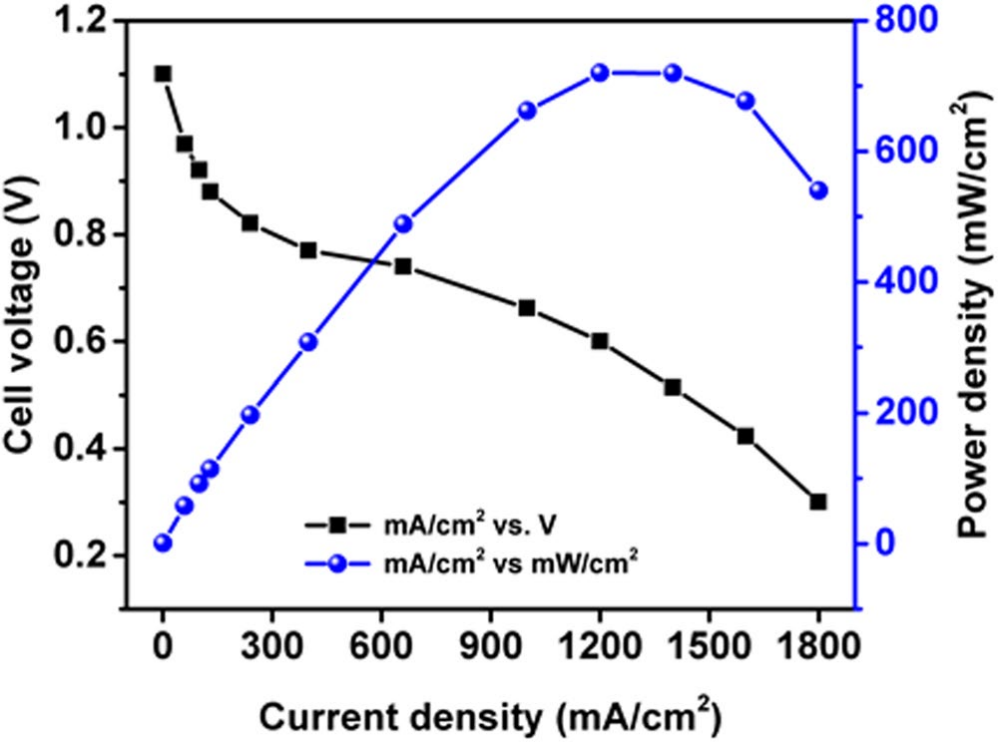
Single cell PEM fuel cell of J&M catalyst with PP substrate.

The MEA is fabricated with PP decal substrates and assembled in 5 cm^2^ active area fuel cell. The I-V performance of the MEA is studied under constant current mode. The fixture conditions are atmospheric pressure, 65 °C, 0.25SLPM of hydrogen and oxygen. At 1.2 A.cm^−2^ current density and 720 mW.cm^−2^ power density a cell voltage of 0.6 V has been observed (refer Fig. 8) that is on par in performance with any commercial MEA’s. The fuel cell performance of MEA from PP film is also compared with other MEAs from different substrates and found to be of superior performance as presented in Table S1.

## Conclusions

Structure property relations of ultra-low cost decal substrates have been scientifically evaluated for MEA fabrication in PEMFC. Across all substrates, PP films have shown 100% catalyst transferability. DSC studies indicate the PP substrate to be thermally stable at hot-press conditions and henceforth there is no poisoning of the catalyst layer. Catalyst ink has good wettability with PP films and exhibit uniform film forming capability. SEM imaging is done to study the surface characteristics of the electrode. Furthermore, the electrochemical studies confirm the performance of PP based MEAs to be on-par with commercial MEAs but at much reduced fabrication cost.

## Materials

Nafion^®^212 polymer electrolyte membrane was obtained from Fuel cell store, Nafion solution (10 wt%)was obtained from Sainergy Fuel cell India PVT.LTD., Serpentine flow channel patterned, single cell PEMFC unit was obtained from Wonatech for Hydrogen-Oxygen test fixture. Carbon paper (Sigracet 35BC) used as gas diffusion layerwas imported from EWII Fuel Cells, 40 wt% Pt-C (Johnson Matthey Hispec-4000) from Sigma Aldrich, 2-propanol (99% purity) from Merck, Polypropylene was procured from Adhesive Specialities, Bangalore. Cellophane tape purchased from Super tape industries, Ahmedabad. Kapton (HN) was procured from Sigma Aldrich. Teflon, Reinforced PTFE procured from Polyfloro limited, Bengaluru. Silicon coated-polymer (Silicone coated PET) was purchased from Loparex India Private Limited, Mumbai. Deionized (DI) water was used throughout the experimentation.

## Methods

### Preparation of catalyst ink slurry

100 mg of JM catalyst was weighed. To it 500 µl water, 1500 µl isopropanol and 300 µl Nafion ionomer were added. The mixture was subjected to stirring for 60 minutes, sonication for 30 minutes and stirring again for 30 minutes.

### Decal coating and MEA fabrication

The decal substrates were placed intact to the glass slide. The catalyst ink slurry was casted over the substrate and drag using a doctor blade with net 210 µm thickness. The catalyst coated decal was allowed to dry at room temperature overnight. The required active area size was cut and weighed before and after hot-press. The final loading of Platinum in the catalyst film was estimated to be 0.4 mg. cm^−2^. The coated substrate was cut to 5 cm^2^ active area and hot-press at 121 °C temperature, 75 kg.cm^−2^ pressure for 3 minutes. After hot-press the catalyst layerwas completely transferred over the membrane.

### Contact angle measurements

Water played an important role in stability and dispersion of catalyst ink over the decal substrate^29^. Therefore, contact angle of the water on substrate studies were conducted using Olympus Polarizing microscope, model no. BX51TRF. The contact angle was measured by link sys integrated software with the instrument. Hydrophilic and hydrophobic analysis was carried on each substrate using contact angle measurement.

### Calorimetric analysis

The temperature stability of each substrate was determined using Digital Scanning Calorimeter Mettler –Toledo, DSC 1 STAR^e^ system at a temperature ramp of 5 °C per minute from 30° to 300 °C and a nitrogen flow rate of 50 mL.min^−1^. All the curves were plotted on origin.

### FTIR measurements

Fourier transform infrared spectroscopy was conducted to know the functional groups of the polymer with the instrument Bruker, Vertex-70/80. The films were placed between NaCl crystals and FTIR was recorded.

### Electrochemical testing

All electrochemical evaluation was conducted using Biologic science instrument Model SP-150. The reactant gases were passed through wash bottles ensuring 100% relative humidity. The whole fuel cell set-up is kept in oven at 65 °C and atmospheric pressure. The mass flow of the reactant gases were controlled by Aalborg mass flow controller. The polarisation curves were studied from constant current and potential modes using built software in test station.

### SEM analysis

FEI Quanta 200 Scanning electrode microscopy was used to investigate the surface electrode morphology of electrode and cross-section of catalyst coated membrane.

### Catalyst layer transfer percentage

The catalyst layer transfer percentage of substrate was calculated using following equation

$$\mathrm{Catalyst}\,\mathrm{layer}\,\mathrm{transfer}\,\mathrm{percentage}=[\frac{\mathrm{Weight}\,\mathrm{of}\,\mathrm{Substrate}\,\mathrm{before}\,\mathrm{hot}\,\mathrm{press}-\mathrm{Weight}\,\mathrm{of}\,\mathrm{substrate}\,\mathrm{after}\,\mathrm{hot}\,\mathrm{press}}{\mathrm{Total}\,\mathrm{weight}\,\mathrm{of}\,\mathrm{catalyst}\,\mathrm{loaded}\,\mathrm{over}\,\mathrm{the}\,\mathrm{substrate}}]\ast 100$$

### Thickness of the substrate

The thickness of the substrate was measured using thickness gauge instrument Mitutoyo thickness gauge.

### Substrate poisoning

The thermal instability of the substrate during hot-press condition poisoned the catalyst that in turn degraded the performance of the MEA. This was referred as catalyst poisoning due to substrate and DSC was conducted to study the thermal stability of all the substrates.

## Electronic supplementary material

Supplementary data
