# Supplementary material for: Studies on structure property relations of efficient decal substrates for industrial grade membrane electrode assembly development in pemfc
Source: Sci Rep. 2018 Aug 14;8:12082. doi: 10.1038/s41598-018-30215-0 (PMC6092413; doi:10.1038/s41598-018-30215-0)
Supplement: Supplementary file 1 — Supplementary data [file 41598_2018_30215_MOESM1_ESM.docx]

**Studies on structure property relations ofefficient decal substrates for industrial grade membrane electrode assembly development in pemfc**

*Sri Harsha Akella, D. Ebenezer, R. S. Sai Siddhardha, Alkesh Ahire, NawalKishor Mal**

*Material Science and Technology, Innovation Center, Tata Chemicals Limited, Pune, India*

**Corresponding author*

| **Substrate Name** | **Thickness of substrate**  **(µm)** | **Catalyst layer transfer**  **(%)** | **Poisoning**  **(**✓**/**🗶**)** | **Scalability**  **(**✓**/**🗶**)** | **Catalyst loading (mg pt. cm^-2^)** | **Constant Current density (A. cm^-2^) applied to get 0.6V** |
| --- | --- | --- | --- | --- | --- | --- |
| PP | 30 | 100 | 🗶 | ✓ | 0.4 | 1.2 |
| LDPE | 30 | 100 | ✓ | 🗶 | 0.4 | 0.2 |
| Si-PET | 50 | 95 | 🗶 | 🗶 | 0.3 | 0.7 |
| Kapton HN | 15 | 92 | 🗶 | ✓ | 0.3 | 0.7 |
| PTFE | 90 | 64 | 🗶 | 🗶 | 0.3 | 0.3 |
| RPTFE | 220 | 72 | 🗶 | 🗶 | 0.2 | 0.7 |

**Table S1.** Comprehensive comparison between different substrates used in the study.
